# Analysis of a real time group consensus peer review process in radiation oncology: an evaluation of effectiveness and feasibility

**DOI:** 10.1186/s13014-018-1190-z

**Published:** 2018-12-03

**Authors:** Ashley A. Albert, William N. Duggar, Rahul P. Bhandari, Toms Vengaloor Thomas, Satyaseelan Packianathan, Robert M. Allbright, Madhava R. Kanakamedala, Divyang Mehta, Chunli Claus Yang, Srinivasan Vijayakumar

**Affiliations:** Department of Radiation Oncology, University of Mississippi Medical Center, 350 W. Woodrow Wilson Drive, Suite 1600, Jackson, MS 39213 USA

**Keywords:** Peer review, Group consensus, Treatment planning, Quality Assurance

## Abstract

**Background:**

Peer review systems within radiation oncology are important to ensure quality radiation care. Several individualized methods for radiation oncology peer review have been described. However, despite the importance of peer review in radiation oncology barriers may exist to its effective implementation in practice. The purpose of this study was to quantify the rate of plan changes based on our group peer review process as well as the quantify amount of time and resources needed for this process.

**Methods:**

Data on cases presented in our institutional group consensus peer review conference were prospectively collected. Cases were then retrospectively analyzed to determine the rate of major change (plan rejection) and any change in plans after presentation as well as the median time of presentation. Univariable logistic regression was used to determine factors associated with major change and any change.

**Results:**

There were 73 cases reviewed over a period of 11 weeks. The rate of major change was 8.2% and the rate of any change was 23.3%. The majority of plans (53.4%) were presented in 6–10 min. Overall, the mean time of presentation was 8 min. On univariable logistic regression, volumetric modulated arc therapy plans were less likely to undergo a plan change but otherwise there were no factors significantly associated with major plan change or any type of change.

**Conclusion:**

Group consensus peer review allows for a large amount of informative clinical and technical data to be presented per case prior to the initiation of radiation treatment in a thorough yet efficient manner to ensure plan quality and patient safety.

## Introduction

The multistep planning process in radiation oncology is a unique practice that often entails subjective decision making and variations among individual radiation oncologists may exist in each step. Therefore, in addition to the inherent systemic errors that are possible in each step, each component of this complex planning process in radiation oncology is also potentially susceptible to human error. As such, strategies to decrease error in the radiation oncology planning process are of great importance for plan quality and patient safety.

In addition to the meticulous quality assurance process that has been established by medical physicists, peer review has been proposed as a strategy to ensure plan quality and patient safety within radiation oncology. Several reports detailing the peer review process in various radiation oncology departments have been published and in these cases, peer review typically involves plan evaluation by a radiation oncologist other than the treating physician as well as feedback from members of a multidisciplinary team such as dosimetrists and medical physicists [1–4]. Additionally, individual radiation oncology departments have also established patient management plans that incorporate published literature, national guidelines, and institutional experience to ensure quality radiation care [5]. Such peer review systems have been shown to result in rates of changes from 3 to 12% and the rate of change varies based on disease site in most cases [3, 6].

A survey of cancer treatment centers in Canada reported that approximately 50% of the centers review 80% of curative-intent plans and another survey demonstrated that 70–80% of radiation plans in the United States undergo peer review and this number may be even lower among non-academic institutions [7, 8]. Furthermore, large variations among departments across the United States may exist in aspects of peer review such as the time dedicated to peer review, the attendance by key personnel, and the extent to which different treatment modalities are reviewed [8]. Despite the importance of peer review in the planning process, barriers may exist to the implementation of effect peer review in individual practices including protected time and scheduling, equipment, and the culture of a department [4, 9, 10].

Similar to other models utilized in radiology, our practice has developed a prospective group consensus peer review model which involves multi-disciplinary review of major treatment plan aspects prior to initial treatment [11, 12]. The aim of this report is to quantify the rate of change of this prospective group consensus peer review process within our individual department as well as the amount of time and resources needed for peer review in order to create a model of group consensus peer review that can be implemented in other radiation oncology departments.

## Materials and methods

Cases that were presented at our institutional department treatment planning conference were prospectively collected over a period of 11 weeks. The total number of patients who underwent Computed Tomography (CT) simulation during this same time period was also recorded. All cases had been discussed previously at “New Patient Conference” at which point treatment intent including dose and technique had been decided upon in the majority of cases with the input of multiple physicians.

As is standard process, the patient will then undergo CT simulation. The organs at risk (OAR) and the treatment volumes are delineated by the resident physician on the planning CT and then approved by the attending physician. The dosimetrist proceeds with planning and the plan is reviewed by the resident and attending prior to presentation in the peer review conference. Per departmental policy, all cases that are considered to be complex (i.e. intensity modulated radiation therapy [IMRT], volumetric modulated arc therapy [VMAT], stereotactic body radiation therapy [SBRT], reirradiation, pediatric, cases that involve adaptive planning or re-planning, or rare cases as determined by the treating physician) must be approved in a multidisciplinary conference prior to the initiation of treatment [2]. These cases are typically presented one to three days prior to the initiation of treatment or on the same day when starting treatment is considered urgent. The remaining cases that are not presented at the treatment planning conference, all of which are treated with three-dimensional conformal radiation therapy (3D-CRT), are presented within one week of starting treatment at the department’s weekly chart rounds. The treatment planning conference is attended by physicians, resident physicians, physic residents, physicists, and dosimetrists. At least two attending physicians other than the treating physician must be present. Our institution’s two campuses are linked via video conferencing and plans are shown simultaneously with the desktop sharing feature of Microsoft Lync.

A synopsis of the patient’s history is provided by the treating physician including information regarding previous chemotherapy or plans for concurrent chemotherapy as well as previous or future oncologic procedures related to the diagnosis. This is then followed by a thorough review of target volumes including gross tumor volume (GTV), clinical target volume (CTV), and planning target volume (PTV). When indicated, additional explanation or rationale regarding decision making used during the generation of target volumes may be provided by the treating physician. During target volume review, any image fusions (i.e. positron-emission tomography [PET]/CT fusion or magnetic resonance imaging [MRI] fusion) performed may also be reviewed. Additionally, any normal tissue contours in close proximity to target volumes in question may be evaluated. After this initial review of volumes, the automated dose volume histogram (DVH) analysis is presented. A scorecard depicts whether an individual dose constraint has been met based on scripted data agreed upon by planners, physicists, and physicians [13]. The color green indicates the dose constraint has been met whereas the color red indicates that the constraint has not been met. Likewise, PTV coverage is also evaluated in the scorecard. Individual DVH’s can then be viewed if dose constraint is not met. The final step in the treatment planning conference is the review of isodose lines in context of the OAR’s, planning risk volumes (PRV), and target volumes. Standard colors are used for the prescription isodose line, 105% isodose line, 95% isodose line, 90% isodose line, etc. Standard procedure involves demonstrating the location of the “hot spot” as well as any concerns related to OAR’s, planning risk volumes (PRV), and target volumes.

During and after presentation of the plan, attendees of the conference are encouraged to make suggestions regarding total dose, dose fractionation, target volumes, beam arrangement, technique, or any other aspect of the treatment plan. To meet the criteria of passing, the plan must be approved by at least two of the non-treating physicians present. If the target volumes are to be modified, the plan is sent back for re-planning. If the dose is increased or decreased, this is changed by the planner in real time and the scorecard is reviewed again with the new dose. If the dose per fraction is changed, re-planning may or may not have to be performed based on the technique used.

In this review, we prospectively collected data for each case presented including the time to present per case, type of case (IMRT, VMAT, 3D), type of patient (adult or pediatric), site of disease, receipt of previous radiation, generation of a composite plan, change in total dose, change in dose per fraction, plan approval or rejection, reason for rejection (change in volume, change in dose), any additional comments about the case, and the number of weekly conferences. Cases were then retrospectively analyzed to determine the rate of major change (plan rejection) and any change in plans after presentation as well as the median time of presentation. Univariable logistic regression was used to determine factors associated with major change and any change. This study did not meet our institution’s criteria for human subject research thus IRB approval was not needed. No protected health information (PHI) was collected for this study.

## Results

We retrospectively analyzed 73 cases over a period of 11 weeks that were presented prior to starting treatment at our institutional department treatment planning conference. A total of 149 patients underwent CT simulation during this same time period. Thus, approximately 49% of our patient plans were considered complex and presented prior to initiating treatment.

We found that our rate of major change for presented cases was 8.2%. A total of 31 treatment planning conferences took place during this period. The most frequently reviewed sites were pelvis (24.7%), followed by lung (23.3%), and head and neck (20.5%). The most common technique utilized in the plans reviewed was VMAT which was performed in approximately 45% of cases presented. The majority of cases presented were adults patients (93.2%). Nearly 18% of presented cases had received previous radiation nearby the area of concern either at our institution or another institution. Composite plans were presented in cases of reirradiation or if a sequential boost was being presented separately from the initial plan. In this series of cases, composite plans were generated in 23.3% of the time. Thirteen percent of plans presented had been previously presented and has undergone some type of recommended modification. A summary of characteristics of cases presented is found in Table 1.

**Table 1:** Summary of characteristics of cases presented

Characteristic	Number of cases (*n* = 73)	Mean time of presentation (minutes)
Site		
Pelvis	18 (24.7%)	8
Lung	17 (23.3%)	11
Head and Neck	15 (20.5%)	9
Abdomen	1 (1.4%)	6
Breast	1 (1.4%)	5
CNS	7 (9.6%)	9
Sarcoma	4 (5.5%)	5
Spine	3 (4.1%)	6
Thorax	7 (9.6%)	9
Modality		
IMRT	13 (17.8%)	9
VMAT	33 (45.2%)	8
Low-mod IMRT	7 (9.6%)	10
SBRT	8 (11.0%)	10
3D-CRT	12 (16.4%)	7
Pediatric		
Yes	5 (6.8%)	10
No	68 (93.2%)	8
Reirradiation		
Yes	13 (17.8%)	9
No	60 (82.2%)	8
Composite plan		
Yes	17 (23.3%)	8
No	56 (76.7%)	9
Representation		
Yes	10 (13.7%)	11
No	63 (86.3%)	8
Length of presentation		
0–5 min	19 (26.0%)	–
6–10 min	39 (53.4%)	–
11–15 min	11 (15.1%)	–
> 15 min	4 (5.5%)	–

*CNS* central nervous system, *IMRT* intensity modulated radiation therapy, *VMAT* volumetric modulated arc therapy, Low-mod *IMRT* low modulated intensity modulated radiation therapy, *SBRT* stereotactic body radiation therapy, *3D-CRT* Three-dimensional conformal radiation therapy

As previously described if the plan in this conference must undergo major modifications such as target volumes changes or dose fractionation changes resulting in re-planning, it is considered rejected. This occurred in 8.2% of the cases. Half of these major changes were due to changes in target volumes and the other half were due to changes in dose necessitating re-plan. The total dose was changed in 16.4% of cases presented and dose per fractionation was changed in 6.8% of cases. Overall, the total rate of change including both changes resulting in re-planning and those that did not result in re-planning was 23.3%. A summary of the change rates is found in Table 2.

**Table 2:** Rates of change of plan after presentation

Type of change	Number of cases (*n* = 73)	Mean time of presentation (minutes)
Total dose		
Yes	12 (16.4%)	10
No	61 (83.6%)	8
Dose per fraction		
Yes	5 (6.8%)	9
No	68 (93.2%)	8
Rejection		
Yes	6 (8.2%)	10
No	67 (91.8%)	8
Any change		
Yes	17 (23.3%)	10
No	56 (76.7%)	8

The majority of plans (53.4%) were presented in 6–10 min. Overall, the mean time of presentation was 8 min. Of the sites presented, the longest mean time of presentation was for lung cases (11 min). This was followed by head and neck (9 min) and thorax (9 min). When categorized by radiation modality, low-modulated intensity-modulated radiation therapy (low-mod IMRT) and stereotactic body radiation therapy (SBRT) had the longest mean time of presentation (10 min). Cases that received previous radiation, had a composite plan generated, or were being re-presented had slightly longer presentation times as compared to those that did not. Additionally, cases that had any type of change also had slightly longer presentations as compared that cases that were approved without any major or minor changes. A summary of the mean time of presentations is found in Table 1 and Table 2.

On univariable logistic regression, volumetric modulated arc therapy plans were less likely to undergo a plan change. Otherwise, there were no factors significantly associated with major plan change or any type of change. A separate univariable logistic regression was performed to identify predictors for any type of plan change. However, no factors that were significantly associated with any plan change were identified. A summary of these data are found in Table 3.

**Table 3:** Univariable logistic regression for any plan change and plan rejection

Any plan change (total change)		
	OR (95% CI)	*p*-value
Pediatric		
No	1	
Yes	2.35 (0.37–15.4)	0.371
Technique		
IMRT	1	
VMAT	0.08 (0.01–0.46)	0.005
Low-mod IMRT	1.56 (0.24–9.91)	0.640
SBRT	0.39 (0.06–2.70)	0.339
3D-CRT	0.39 (0.07–2.10)	0.277
Reirradiation		
No	1	
Yes	2.50 (0.69–9.02)	0.162
Composite plan		
No	1	
Yes	2.23 (0.67–7.35)	0.187
Representation		
No	1	
Yes	2.56 (0.63–10.4)	0.189
Site		
Pelvis	1	
Lung	3.50 (0.73–16.8)	0.118
Head and Neck	0.77 (0.11–5.34)	0.791
Abdomen	0.01 (0.01–2.30)	0.990
Breast	8.07 (0.01–10.0)	0.990
CNS	0.01 (0.01–9.00)	0.999
Sarcoma	1.67 (0.13–22.0)	0.698
Spine	0.01 (0.01–1.01)	0.999
Thorax	3.75 (0.54–26.1)	0.183
Major plan change (plan rejection)		
	OR (95% CI)	p-value
Pediatric		
No	1	
Yes	3.15 (0.29–33.7)	0.343
Technique		
IMRT	1	
VMAT	0.38 (0.02–6.48)	0.500
Low-mod IMRT	2.00 (0.11–37.8)	0.644
SBRT	1.71 (0.09–31.9)	0.718
3D-CRT	2.40 (0.19–30.5)	0.500
Reirradiation		
No	1	
Yes	917 (0.10–8.58)	0.939
Composite plan		
No	1	
Yes	0.638 (0.07–5.87)	0.691
Representation		
No	1	
Yes	1.29 (1.35–12.3)	0.826
Site		
Pelvis	2.27 (0.19–27.5)	0.521
Lung	1.21 (0.07–21.2)	0.894
Head and Neck	0.02 (0.01–1.01)	0.999
Abdomen	2.74 (0.01–3.00)	0.999
Breast	0.01 (0.01–1.01)	0.999
CNS	0.01 (0.01–1.01)	0.999
Sarcoma	0.01 (0.01–1.01)	0.999
Spine	0.01 (0.01–1.01)	0.999
Thorax	2.83 (0.15–52.7)	0.485

## Discussion

In this single institution review of peer-reviewed cases, we found a major change rate of 8.2%, a total change rate 23.3%, and a mean presentation time of 8 min per case. We found this to be consistent with other reported rates of change and time per presentation [3, 6, 14].

Within the field of radiology, consensus-oriented peer review (COGR) has been developed to improve upon the limitations of previous peer-review systems such as nonrandom selection of cases, lack of anonymity of the reviewee, length of the process, and limited participant feedback [11]. In the context of radiology, COGR entails groups of radiologists discussing cases in a conference setting and a consensus is reached about whether the report is acceptable or should be modified. This method allows multiple questions to be incorporated into the feedback process and allows for real time feedback. Additionally, this system is felt to foster a culture of safety in which radiologists can openly discuss issues related to reports [12]. We have found that a similar method of group consensus peer review with radiation oncology offers some of these same advantages for radiation therapy plan review including real time feedback that can be incorporated into the planning process. Although the treating physician in our group consensus peer review process not anonymous, the fact that all complex plans must be approved in conference cultivates a culture in which peer review is an integral and routine part of the planning process.

Several different models have been suggested for the peer review process within radiation oncology. Some practices have established have site specific processes such as the inclusion of physical exams by additional physicians or image review by radiologists that may alter contours [15, 16]. Another example of individualized peer review is the incorporation of a prospective contouring rounds which feedback about contours and modification takes place prior treatment planning [17]. Given the variations in set up and clinic flow, individual departments may need to base their format of peer review on the unique operations of their clinic with certain considerations such as the number and type of patients treated, the number of campuses, and other educational conferences, etc. In our experience, we found that a routine conference in which all treating physicians are presenting all aspects of their cases in one setting and plans are then modified when indicated based on group consensus prior to treatment initiation allows for an effective peer review process.

Furthermore, it may be important for radiation oncology clinics attempting to integrate an effective peer review process within their department to have an estimate of the time needed for such a process. We found that although one hour time blocks were scheduled twice weekly, we actually held conference closer to an average of three times per week. Some conferences were held urgently at lunch time so as not to delay care for case that needed to start. We found that with some built in flexibility, we were able to accommodate such instances and prevent delays in care. This information may be useful for scheduling time for peer review and preventing barriers to this important process.

Additionally, we identified some differences in the time needed to present cases based on the site of involvement and the modality utilized. In our experience, cases involving lung cancer had the longest time of presentation. Congruently, SBRT and low-modulated IMRT which we use to treated our limited stage lung cancer and locally-advanced lung cancer respectively had the longest time of presentation among the modalities utilized. In the case of SBRT, more time may be used to carefully review the isodose lines near critical structures as well as the coverage of the target volumes due to the higher dose per fraction as compared to non-SBRT plans. In our cases of locally-advanced lung cancers, we are often treating very large tumors and the plans therefore frequently exceed recommended dose constraints for the lungs and heart. In these cases, the total dose may be reduced or recommendations may be made adaptive planning or even for induction chemotherapy followed by re-simulation to assess for decrease in the size of the tumor. We also found that cases involving the central nervous system (CNS) and head and neck involved slightly more time likely due to the higher number of organs at risk that are reviewed for these sites. Finally, cases in which some type of change was recommended had slightly longer total times of presentation which likely accounts for the additional discussion that took place during the presentation of these cases.

In this series, 18% of cases presented involved patients who had received previous radiation in an area in close proximity to the current area of concern. In the majority of these cases, MIM software was utilized to create a composite plan with previous doses. Some of these cases received previous radiation at an outside facility whereas some received radiation at our institution. This conference allowed for important feedback in cases that may be considered high risk for toxicity and therefore proves to be an effective method of ensuring patient safety especially for those that have received previous radiation.

Although we identified some differences in the type of cases presented at this conference and the time needed for presentation based on site, modality, and complexity of the plan, we found no significant predictors for major plan change or any type of change other than categorization of VMAT plans. VMAT plans were less likely to undergo any type of change or rejection. We attribute this to the large number of prostate plans which are planned with VMAT and typically meet all dose constraints. The fact that there were no other significant predictors is noteworthy as it illustrates that changes may or may not be made to plans based on feedback from the peer review process for any type of case but such changes may be difficult to systematically predict. For example, the treating physician may consider the presented cases to be “straight-forward” or not complex and unlikely to undergo any change prior to treatment initiation. However, after review from colleagues during the peer review process, additional viewpoints may bring to light unbiased considerations. As such, a systematic process which allows for peer review to place on a regular basis for plans prior to the initiation of treatment is key for radiation plan quality and safety.

In this series, we found a major plan change rate of 8.2% which is in alignment with the change rate reported for many other peer review processes [3, 6]. In addition to major plan changes, we also tracked any type of minor change including change in total dose or dose per fraction that may not have necessitated re-planning but did represent a modification based in the peer review process. We found that we had a total rate of change of 23.3%. We considered this to be another significant point as it demonstrates how the peer review process can result in seemingly small and detailed changes that may still impact the outcome of the radiation plan that otherwise would not have been made without the peer review process.

In addition to quality peer review, this treatment planning conference allows for additional educational opportunities outside of other structured teaching settings. Resident physicians are exposed to the plans of multiple treating physicians and hear the feedback that is provided in the conference. They are also encouraged to provide their own feedback and ask questions. Furthermore, they can become familiar with technical challenges that may be present in planning and become proficient in plan evaluation. Resident physicians are encouraged to review the plans with the dosimetrist prior to presentation in conference and as a result they become familiar with the scorecard results from the automated DVH analysis. They then can take an active part in the peer review process by providing additional information about the case when indicated. Likewise, physics residents who participate in the planning process are encouraged to present the cases that they helped plan. Finally, given the variety of the type of cases presented, each attending physician can stay abreast of pearls of treatment for that site even if it is not their main site of focus. Therefore, in addition to ensuring safe care for those patients whose cases are presented, this peer review process also educates both trainees and seasoned physicians thereby contributing to the quality of radiation treatment delivered for future patients. Furthermore, although not a standard part of our current peer review process, some institutions incorporate comparison data from previous plans as an additional aspect of evaluating a plan. Such a process compares metrics such as select DVH values from the plan under review to those from all previous plans of the same site. This continual comparison would allow a department to be aware of their current planning capabilities and continue to work towards improvement in the planning process.

Finally, as the field of radiation oncology continues to move forward, advancements from emerging technologies such as artificial intelligence (AI) may further enhance the process of peer review. AI has the potential to allow physicians to perform an even more detailed analysis of all aspects of radiation plans in less time [18]. For example, algorithms that could take into account not only expert opinions regarding target delineation but also treatment outcomes based on radiomic signatures as well as probabilities of normal tissue complications would help move peer review from a potentially subjective process to a completely evidence-based approach to quality assurance.

## Conclusions

We conclude that through the process of group consensus peer view modeled in our radiation oncology clinic, a large amount of informative clinical and technical data can be presented per case prior to the initiation of radiation in a thorough yet efficient manner in a collegial environment open to feedback in order to ensure that radiation treatment is safe and effective for our patients.

## Abbreviations

AI: Artificial Intelligence
CNS: Central nervous system
COGR: Consensus-oriented peer review
CT: Computed Tomography
CTV: Clinical target volume
DVH: Dose volume histogram
GTV: Gross tumor volume
IMRT: Intensity Modulated Radiation Therapy
MRI: Magnetic resonance imaging
OAR: Organs at risk
PHI: Protected health information
PRV: Planning risk volume
PTV: Planning target volume
SBRT: Stereotactic body radiation therapy
VMAT: Volumetric modulated arc therapy
